# *Mycobacterium tuberculosis* EspR modulates Th1-Th2 shift by transcriptionally regulating IL-4, steering increased mycobacterial persistence and HIV propagation during co-infection

**DOI:** 10.3389/fimmu.2023.1276817

**Published:** 2023-10-19

**Authors:** Sriram Yandrapally, Anushka Agarwal, Archismita Chatterjee, Satarupa Sarkar, Krishnaveni Mohareer, Sharmistha Banerjee

**Affiliations:** Department of Biochemistry, University of Hyderabad, Hyderabad, India

**Keywords:** *Mycobacterium tuberculosis*, EspR, Th1 immune response, Th2 immune response, cytokines, gene regulation

## Abstract

*Mycobacterium tuberculosis* (*Mtb*) and HIV are known to mutually support each other during co-infection by multiple mechanisms. This synergistic influence could be either by direct interactions or indirectly through secreted host or pathogen factors that work in trans. *Mtb* secretes several virulence factors to modulate the host cellular environment for its persistence and escaping cell-intrinsic immune responses. We hypothesized that secreted *Mtb* transcription factors that target the host nucleus can directly interact with host DNA element(s) or HIV LTR during co-infection, thereby modulating immune gene expression, or driving HIV transcription, helping the synergistic existence of *Mtb* and HIV. Here, we show that the *Mtb*-secreted protein, EspR, a transcription regulator, increased mycobacterial persistence and HIV propagation during co-infection. Mechanistically, EspR localizes to the nucleus of the host cells during infection, binds to its putative cognate motif on the promoter region of the host IL-4 gene, activating IL-4 gene expression, causing high IL-4 titers that induce a Th2-type microenvironment, shifting the macrophage polarization to an M2 state as evident from CD206 dominant population over CD64. This compromised the clearance of the intracellular mycobacteria and enhanced HIV propagation. It was interesting to note that EspR did not bind to HIV LTR, although its transient expression increased viral propagation. This is the first report of an *Mtb* transcription factor directly regulating a host cytokine gene. This augments our understanding of the evolution of *Mtb* immune evasion strategies and unveils how *Mtb* aggravates comorbidities, such as HIV co-infection, by modulating the immune microenvironment.

## Introduction

HIV and tuberculosis (TB) are the two most devastating infectious diseases that are emerging as a syndemic. There is no cure for HIV and the anti-retroviral therapy only prolongs the life expectancy with severe side effects. The co-infection of HIV and TB spirals the complications leading to a fatal outcome. While *Mycobacterium tuberculosis* (*Mtb)* primarily infects and replicates in host macrophages and dendritic cells, HIV has a wider host tropism (1). HIV infection gradually declines the CD4^+^ T cells leading to a sharp decline in immune responses (2). The decline in CD4^+^ T cells is a conducive state for the activation of TB (3), the most common secondary infection in HIV-infected patients. The influence of each other during co-infection could be mediated either by direct interaction of host and pathogen factors or indirectly through secreted host or pathogen factors that work in trans. Many *Mtb* factors have been associated with augmenting HIV titers (4, 5), however, the molecular mechanisms behind the impact are not clear for all the factors.

*Mtb* secretes several proteins towards adaptation to hostile microbicidal stress conditions. Some of these proteins also positively regulate HIV LTR and therefore viral replication (6, 7). One of the secreted proteins of the *Mtb* ESX-1 secretory system, EspR, is a transcription factor that controls its virulence (8). Besides this, EspR also regulates the ESX-1 operon by binding to the upstream espACD locus (9) and is secreted out by its transcriptional targets, making it unavailable for further activation, and exhibiting a negative feedback mechanism (10). Raw 264.7 (a murine macrophage cell line) stably expressing EspR was shown to inhibit BCG-induced apoptosis by intercepting the MyD88 pathway, indicating the role of EspR in counteracting host-induced innate defense (11). Although the role of EspR in *Mtb* gene regulation is well understood, its role in host-pathogen interaction, when secreted in the host milieu, is limited.

Many mycobacterial surface components and secreted proteins interact with host-pathogen recognition receptors, such as TLR2, polarizing the host macrophages to the M1 state. The cytokines released by the M1 polarized macrophages are pro-inflammatory, which brings about a Th1-type immune response, responsible for cell-mediated immunity (12). The clearance of *Mtb* and HIV-infected cells requires a Th1-dominant environment that activates effector T cells and cytotoxic T cells, the failure of which has been associated with both HIV and TB disease progression (13, 14). In response to the hostile intracellular conditions, virulent mycobacteria secrete several proteins shifting the polarization of macrophages to an alternate or compromised repair state M2 (15). The cytokines released by macrophages in the M2 state favor a Th2-type immune response, promoting bacterial persistence and HIV replication by dampening the host defenses (16, 17). We and others have reported that TB patients and those with HIV co-infection have mixed Th1/Th2 responses with elevated levels of both the kind of cytokines (18–20). The molecular mechanisms and participation of mycobacterial factors behind such mixed responses are still elusive.

One of the ongoing studies of our laboratory is directed towards understanding the role of secreted *Mtb* transcription factors in mycobacterial pathogenesis and HIV-TB co-infection. In the present study, we shortlisted the transcription factors (**Table 1**) from the list of experimentally verified *Mtb* culture filtrate proteins (21). Among the shortlisted transcription factors, we focused on EspR and explored its functional role in the host macrophages. Here, we hypothesized that since EspR is a transcription factor, and if it gains access to the host nucleus, it could modulate host gene expression. In the present study, we found that EspR enters the host nucleus and commandeers the IL-4 gene expression by binding to its upstream promoter, leading to a Th2-type immune response and thereby increasing mycobacterial survival. We also observed that transiently expressing EspR or infection with EspR over-expressing mycobacteria in the background of HIV infection supported increased viral propagation, thereby adding new information to the role of mycobacterial factors in co-infection biology.

**Table 1:** Shortlist of possible *Mycobacterium tuberculosis* secreted transcription factors.

Transcription factor	Target gene (s)	Structure
Rv3849 (espR)	espACD	Helix turn helix
Rv3133c (DevR)	hypoxic response genes	Helix turn helix
Rv0022 (WhiB5)	sigM, ESX-2 and ESX-4	Helix turn helix
Rv0078	Rv0077	Helix turn helix
Rv0472c (TetR-family)	–	Helix turn helix
Rv0903c (prrA)	–	Helix turn helix
Rv2258c	–	Helix turn helix
Rv3246 (mtrA)	dnaA gene	Helix turn helix

The symbol “-” indicates information not available.

## Methodology

The experiments were performed as per the approved protocols by the institutional biosafety committee of the University of Hyderabad, Hyderabad, Telangana, India.

### Mycobacterial culture and HIV-1 strains

*Mtb* H37Rv and *Mycobacterium smegmatis* were grown either in 7H9 medium supplied with OADC (standard growth condition) or in Sauton’s minimal medium with acidic stress (pH 5.5), or oxidative stress (10 mM H_2_O_2_), or hypoxia (22–24) or nutrient starvation stress (grown in PBS) (25). Any possible contamination in *Mtb* culture was checked by Zeil-Neilson staining kit (HIMEDIA, India). HIV-1 strains used in the study include NL4.3 (X4 virus) or NL-ADA8 (R5 virus) (26–29).

### Cell lines and antibodies

HEK293T cell line was maintained in DMEM (HIMEDIA, India) and THP-1 cell line (30) in RPMI-1640 (HIMEDIA, India) media, supplemented with 10% Fetal Bovine Serum (Gibco, USA), 100 U/ml of penicillin, and 100 ug/ml of streptomycin (HIMEDIA, India) at 37°C with 5% CO_2_ (31).

Different antibodies used to monitor the expression and localization of various proteins include anti-Myc antibody (Santacruz biotechnology (sc40); GAPDH (Santacruz biotechnology (sc47724); GFP antibody (Santacruz biotechnology, sc-9996 and ABclonal, AE011); anti-His antibody (Santacruz biotechnology, sc-8036), CD206 antibody (Santacruz biotechnology, sc-58986 PE), CD64 antibody (Santacruz biotechnology, sc-1184 FITC) and secondary antibodies include anti-mouse HRP (cell signaling, 08/2017), anti-rabbit HRP (Santacruz biotechnology, sc-2357) or anti-mouse Alexa 647 (Ab150115). The quantification of HIV for infection assays was performed using HIV-1 p24 Capture ELISA Assay Kit (ABL Inc, USA) and the released HIV antigens were measured using Microlisa HIV Ag & Ab assay kit (detects gp41, gp120, and p24) (JMitra &Co Pvt Ltd, India).

### Transfection

THP-1 monocytes were differentiated into macrophages by treatment with PMA (Sigma, USA) (100 ng/ml) and incubated for 24 h. Subsequently, the cells were incubated in fresh media until macrophage morphology was observed (~24 h). For transfection, 6-8 x 10^5^ HEK 293T cells or THP-1 macrophages were seeded in a 6-well plate 12-16 h before transfection. Fresh media was replaced before transfection with DNA complexes made using either Lipofectamine 2000 (Invitrogen, USA) (for THP-1 cells) or calcium phosphate (SIGMA, USA)(for HEK293T) (32) for about 6 h and used for experimental analyses 48 h post-transfection.

### Plasmid constructs and recombinant EspR purification

The EspR ORF was amplified from *Mtb* H37Rv genomic DNA and cloned in pCDNA3.1 using BamH1 and XhoI sites (pC-EspR). pET-EspR was generated by cloning EspR ORF in pET28a using BamH1 and XhoI. pMSP12::mCherry-EspR was generated by cloning EspR ORF in pMSP12::mCherry (procured from the Addgene #30169) using KpnI and HindIII sites. pET-EspR transformed *E. coli* BL-21λDE3 (Agilent technologies, USA) cells were used for purification of recombinant *Mtb*EspR protein (carrying N and C terminal 6X histidine tag) upon induction with 1 mM Isopropyl β-D-1-thiogalactopyranoside (IPTG) (Fermentas, USA) for 4 h at 37°C under native conditions using Talon-affinity resin (Clontech, USA) according to the manufacturer’s protocol and eluted with 200 mM imidazole containing PBS. Subsequently, r*Mtb*EspR was dialyzed against PBS supplemented with 50 mM NaCl, and 10% glycerol. The protein concentration was measured using 1X Bradford Reagent (Bio-Rad, USA) according to the manufacturer’s instructions and the integrity of the protein was checked on 15% SDS PAGE (**Supplementary Figures S1A, B**).

### Anti-EspR antibody generation

Anti-EspR antibody was raised as per protocol approved by the Institutional Animal Ethics Committee (UH/IAEC/SB/2022/45), by injecting 500 µg purified r*Mtb*EspR diluted in a mixture of 0.5 ml PBS and 0.5 ml complete Freund’s adjuvant into a healthy rabbit as a prime dose. A booster dose was scheduled every two weeks with a gradual decrease (by half) of r*Mtb*EspR protein and emulsified with incomplete Freund’s adjuvant and the cycle was repeated five times. At the end of the schedule, approximately 10 ml of blood was collected, and serum was isolated by centrifugation at 2000 x g for 10 min and confirmed by western blotting with purified protein including a non-specific protein as a negative control (**Supplementary Figure S1C**).

### Electrophoretic mobility shift assay

EMSA was carried out as described with minor modifications (33). Briefly, the chemically synthesized EspR cognate sequences (FL, ΔS1, ΔS2) (IDT/Eurofins, Germany) were annealed and radiolabeled with γP^32^ using T4 PNK (NEB, England). The binding assays of recombinant EspR to different probes were performed in NEB 2.1 buffer supplemented with 50 ng/ml pdIdC, 10% glycerol, and in the presence or absence of unlabeled molar excess of cognate DNA (cold/homologous competition) for 30 min at ambient temperature (25-30°C). The DNA-protein complexes were resolved on a 5% native acrylamide gel (39: 1) at 200 V in 0.5 X TBE. Subsequently, the gels were dried and exposed to a phosphor imaging screen overnight (16-20 h) and imaged using a Typhoon scanner (BIO-RAD, USA) with Quant One software.

### Infection and persistence

For infection, THP-1 macrophages were incubated with wildtype or transformed *M. smegmatis* at an MOI of 20 to 25 for 4 h. Subsequently, the cells were washed with PBS and replaced with fresh media. For bacterial persistence, the infected THP-1 cells were lysed in 100 ul water (per 10^4^ cells) for 30 min at 37°C in 50 ul duplicates. Each aliquot was either processed by the Alamar blue method or plated on 7H10 plates and incubated over 4-5 days. The resulting colonies were counted and the cfu was calculated using the formula cfu/ml= no of colonies * dilution factor/volume of the culture plated. 100 ng/ml of p24 equivalent of HIV-1 (produced from pNL4.3 or NL-ADA8) was used for infection assays in the presence or absence of *Mtb*EspR as described in the results section. The comparative release of viral antigens was quantified after 48 h and expressed as a relative percentage compared to the control experiment.

### Immunofluorescence microscopy

HEK293T cells were washed twice with PBS (HIMEDIA, India) and fixed with 4% formaldehyde (Sigma, USA) for 20 min. The fixed cells were again washed with PBS to remove formaldehyde and subsequently permeabilized by using 0.5% Triton-X-100 (Qualigens, India) for 15 min followed by washing the cells twice with PBS and then blocking with 3% BSA (SRL, India) for 30 min. The cells were washed once with PBS and incubated in the appropriate primary antibody (in 1% BSA) for 2 h, followed by three washes with PBS. The cells were further incubated with the corresponding secondary antibody tagged with Alexa 647 (Ab150115) for 1 h. Subsequently, the cells were washed with PBS and the nuclei were stained with DAPI (Abcam, USA). Imaging was done by Leica confocal microscopy and visualized by Las X software. Plot profiles were generated by using Image J and Huygens software.

### Live cell imaging

Around 0.3 million infected THP-1 cells (with *M.smeg::pMSP12* or *M.smeg::Mtb*EspR) were seeded in a live cell imaging dish (Ibidi GmbH, Germany). The cells were treated with Hoechst dye (Thermo Scientific, USA) for 15 min. Later, the cells were washed twice with PBS and finally replaced with fresh media and subjected to live cell imaging (Leica confocal microscope). The images were collected for 30 min to 1 h for every 30 s and visualized by Las X software.

### Western blotting

*Mtb* H37Rv cultures grown under different stress conditions were used to harvest both cells and supernatant. The cells were resuspended in PBS and lysed using a bead beater for 10-20 cycles of 1 min pulse and 2 min pause. The culture supernatant was passed through a 0.2 μm filter. The protein concentrations (of both cell lysates and culture filtrate) were quantified by Bradford assay. Subsequently, the samples were fractionated on SDS-PAGE and transferred onto a nitrocellulose membrane (PALL, BioTrace™ NT nitrocellulose, USA) and confirmed by ponceau (Fluka Analytical, USA) staining. The blots were blocked with 5% skimmed milk (HIMEDIA, India) for 1 h at room temperature, followed by 3 washes with PBS and then incubated with primary antibody overnight at 4°C. The next day, the blot was washed thrice with PBS and further incubated with appropriate secondary antibody for 2 h and developed using a WesternBright™ ECL kit (Advansta, K-12045-D20) in a Biorad ChemiDoc instrument. The secreted levels of EspR were quantified by densitometry using Image J and represented in terms of fold change normalized to the total protein loaded in each condition.

### RNA extraction and qRT-PCR

To measure the mRNA levels of IL-4 in THP-1 cells, the RNA was isolated by using a GeneJET RNA purification kit (Thermo Scientific, USA) according to the manufacturer’s protocol. For mycobacterial RNA, *Mtb* H37Rv cultures grown under different conditions were harvested and resuspended in Trizol (Invitrogen, USA) and lysed using a bead beater for 15 cycles of 1 min pulse and 2 min pause. The lysate was centrifuged at high speed for 10 min at 4°C and processed for RNA isolation following the manufacturer’s protocol. Further, 10 µg of isolated RNA was treated with DNase I (NEB, England) to remove any genomic DNA contamination. 1ug of DNase I treated RNA was converted to cDNA by using the iScript cDNA synthesis kit (BIO-RAD, USA) according to the manufacturer’s protocol. The diluted cDNA was used as the template for the qRT-PCR. The qRT-PCR was performed using the iTaq Universal SYBR green Supermix (BIO-RAD, USA) following the manufacturer’s protocol, in a 96-well plate (Applied Biosystems, USA) and analyzed using QuantStudio3 software.

### Luciferase assay

HEK 293T or THP-1 cells transfected with appropriate plasmids were maintained as described for 48 h. The cells were washed with PBS and harvested by trypsin treatment (0.25 X for 1 min at 37°C) and again washed with PBS. The cells were lysed and processed for luciferase assay (Promega Luciferase Assay system, USA). Briefly, the samples were incubated at room temperature for 20 min followed by a brief vortex. The samples were frozen at -80°C for 1 h, freeze-thawed for 2 more cycles, and centrifuged at 11,000 rpm for 5 min at 4°C. The supernatant was collected in a fresh microfuge tube and the luminescence was recorded using the modulus single-tube multimode reader (Promega) according to the manufacturer’s protocol.

### TZM-bl assay

TZM-bl cells, a cell line with a β-galactosidase reporter gene driven by HIV LTR (34), were infected with equal volume of culture supernatants collected from HEK293T cells transfected with either pCDNA or pC-EspR along with NL-ADA8 as described (35). Briefly, after 24 h of infection, the cells were washed with cold PBS (HIMEDIA, India) and harvested upon trypsin treatment. Cells were centrifuged at 1000 rpm for 5 min and washed once again with PBS and processed as described above.

### Flow cytometry

HEK 293T or THP-1 cells transfected with appropriate plasmids were harvested by trypsin treatment and washed with PBS thrice. The cells were passed several times through a blunt 20-gauge needle fitted to a sterile syringe, to make a single-cell suspension (without any visual clumps). These cells were processed for immunostaining and acquired on an S3e cell sorter (FACS) to evaluate the cell surface markers for M1 and M2 polarization states and the data was analyzed using FACS Express 6 software.

### Statistical analysis

Plots were generated and the statistical significance was determined by a two-tailed unpaired student’s t-test in GraphPad Prism. A p-value <0.05 was considered statistically significant. All the experiments were performed at least thrice.

## Results

### *Mtb*EspR is differentially expressed and is secreted during microbicidal stress conditions

**Figure 1:**
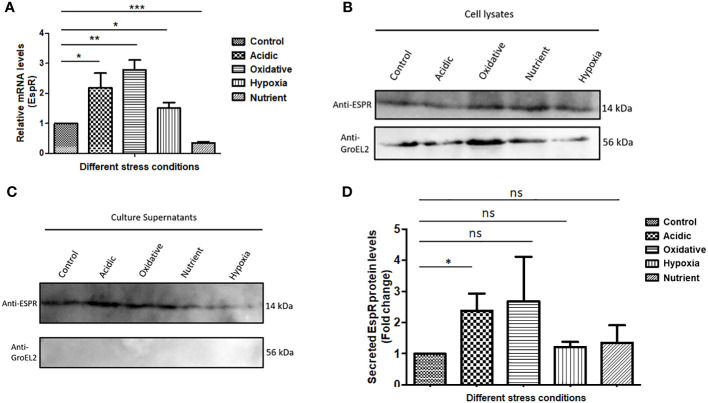
*Mtb*EspR is differentially expressed and secreted during abiotic stress conditions **(A)** The levels of EspR RNA from *Mtb* H37Rv were measured in microbicidal stress conditions acidic, oxidative, hypoxia, and nutrient starvation as compared to standard growth conditions by qRT-PCR. **(B)** Immunoblots showing EspR protein levels in the microbicidal stress conditions in *Mtb* H37Rv cell lysates. GroEL2 was used as a lysis control. An equal amount (as measured by Bradford assay) of lysates was loaded in each well **(C)** Immunoblots showing the EspR protein levels in the respective culture supernatants. **(D)** Bar graph showing immunoblot-based quantification of EspR secretion for experiments represented in **(C)** The quantification for each condition was normalized to the total protein loaded. All the experiments were done at least 3 times. The significance was determined using an unpaired student’s t-test. The p-values are denoted as ***p <= 0.001; **p <= 0.01; and *p <= 0.05, while non-significant values are denoted by n.s.

As it is known that *Mtb*EspR is secreted, we confirmed the same in our experimental conditions. We investigated if it is differentially regulated under conditions mimicking intracellular microbicidal stresses. Towards this, *Mtb* was grown in well-established *in vitro* growth conditions including acidic stress, hypoxia, oxidative stress, and nutrient starvation conditions besides standard growth conditions. Total RNA was extracted from the cultures of each condition and the expression of *espR* RNA was analyzed by qRT-PCR. We observed an increase in the transcript levels of *espR* in acidic (~2-fold), oxidative (~3-fold), and hypoxic (~1.5-fold) conditions as compared to standard growth conditions (**Figure 1A**). Besides, we also investigated the protein levels of EspR, both in the cell lysates and culture filtrates, under these conditions (**Figures 1B, C**). We observed that EspR was secreted in all conditions but was elevated significantly in the culture filtrates of acidic stress as compared to standard growth conditions (**Figures 1C, D**). The levels of EspR, both at transcript and protein levels, were relatively low during nutrient starvation. Overall, these observations confirmed that EspR is secreted both during standard growth conditions and during abiotic stresses.

### *Mtb*EspR supports the survival of mycobacteria and HIV propagation during co-infection

**Figure 2:**
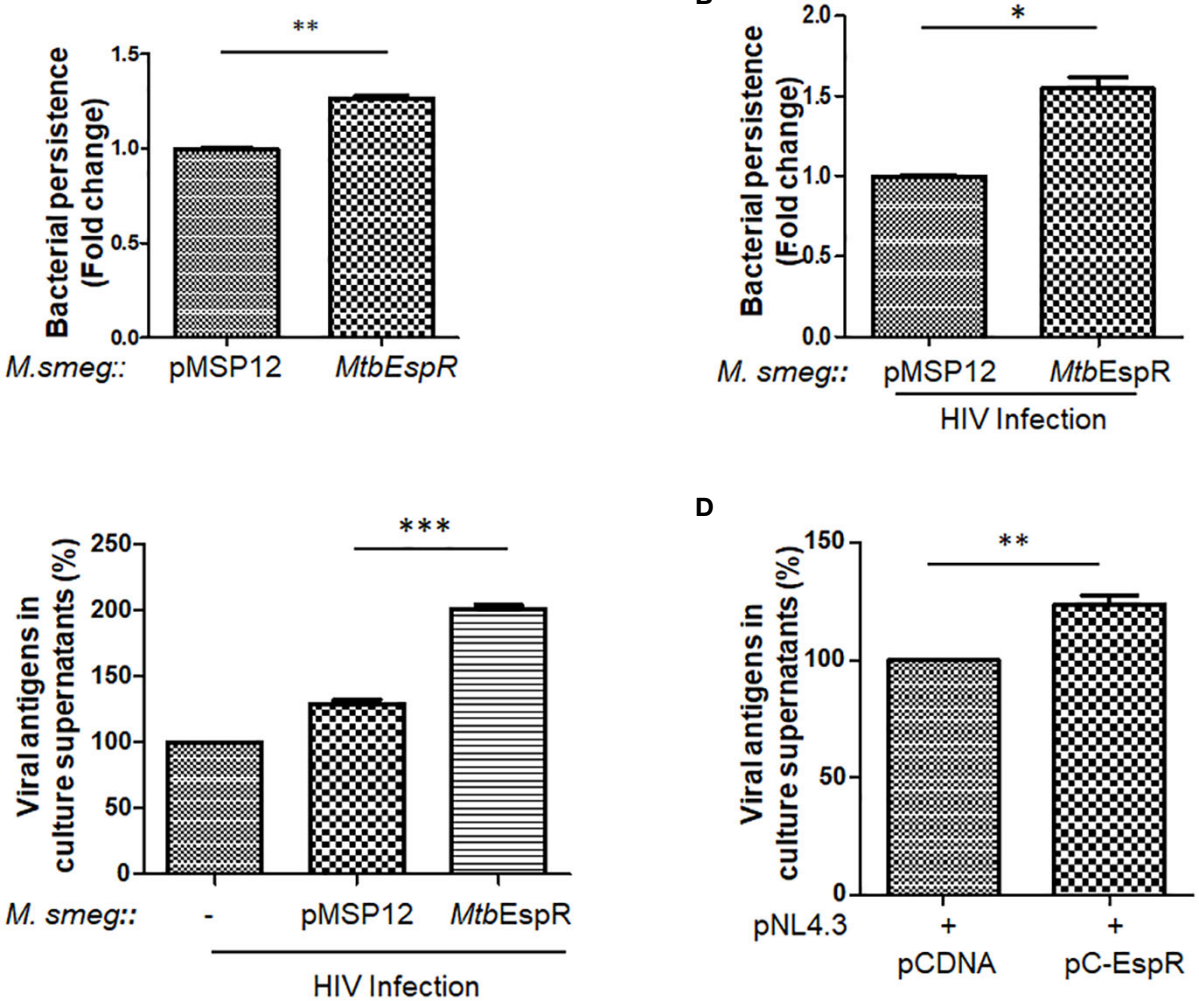
*Mtb*EspR supports the survival of mycobacteria and HIV propagation in host macrophages **(A)** Fold change in the persistence of *M.smeg*::*pMSP12* and *M.smeg*::*Mtb*EspR strains as analyzed in THP-1 cells by Alamar Blue assay **(B)** Fold change in the persistence of *M.smeg*::*pMSP12* and *M.smeg*::*Mtb*EspR strains as analyzed in HIV infected THP-1 cells by CFU **(C)** Relative change in release of viral antigens in the culture supernatant as determined by ELISA in NL4.3, NL4.3+*M.smeg*::*pMSP12*; and NL4.3+*M.smeg*::*Mtb*EspR infection of THP-1 cells, expressed in percentage **(D)** Relative change in release of viral antigens in the culture supernatant as determined by ELISA in pCDNA, pC-EspR transfected HEK293T cells (expressed in percentage). All the experiments were done at least 3 times. The significance was determined using an unpaired student’s t-test. The p-values are denoted as ***p<= 0.001; **p<= 0.01 and *p <= 0.05.

With increased expression and secretion of EspR under stress conditions, we investigated if over-expression of this protein provides an intracellular survival advantage. To prove the same, *Mtb*EspR was ectopically expressed in *M. smegmatis*, an *Mtb* surrogate system, which has limited persistence inside macrophages and is generally cleared by macrophages within 4-9 h during *in vitro* experimental conditions (36). We generated two recombinant strains *M. smegmatis::pMSP12* (vector control, from now onwards *M.smeg::pMSP12*) and *M. smegmatis*::*Mtb*EspR (*M.smeg::Mtb*EspR). The expression and secretion of *Mtb*EspR, which is tagged with mCherry, were verified both in the cell lysates and culture supernatants by western blotting (**Supplementary Figures S2A, B**). The two strains showed no difference during *in vitro* growth kinetics in 7H9 media (**Supplementary Figure S2C**). After confirming secretion of *Mtb*EspR from the recombinant *M. smegmatis* strains under *in vitro* growth conditions, THP-1 cells were infected with *M.smeg::pMSP12* and *M.smeg*::*Mtb*EspR, wherein we observed a 24% increase in the levels of *M.smeg*::*Mtb*EspR than *M.smeg::pMSP12*, suggesting a higher survival of *M. smegmatis* over-expressing *Mtb*EspR (**Figure 2A**). With numerous clinical reports showing the synergistic impact of secondary mycobacterial infections on HIV titers in co-infected patients (18), we further evaluated the effect of *Mtb*EspR on mycobacterial survival and viral propagation during co-infection. THP-1 macrophages infected with HIV NL4.3 were subsequently infected with either *M.smeg::pMSP12* or *M.smeg*::*Mtb*EspR. As reported by us and others, we observed that mycobacterial survival was enhanced in HIV infection background. However, the persistence of *M.smeg*::*Mtb*EspR increased over *M.smeg::pMSP12*, pointing to the role of *Mtb*EspR in mycobacterial survival during co-infection (**Figure 2B**). Similarly, we also evaluated the release of viral proteins in culture supernatants during co-infection, wherein we observed an increase in viral proteins in the culture supernatant upon *M.smeg*::*Mtb*EspR co-infection compared to *M.smeg*::*pMSP12* (**Figure 2C**). To confirm that *Mtb*EspR indeed influences HIV propagation, we transiently expressed *Mtb*EspR in HEK293T cells. The released viral proteins were measured in the culture supernatant 48 h post-transfection by ELISA. We observed a ~24% increase in released viral proteins upon transient expression of *Mtb*EspR (**Figure 2D**) as compared to vector control. Similarly, we performed experiments with the M-tropic (R5) virus of NL-ADA8 to verify the effect of *Mtb*EspR on R5 viruses. We observed a similar increase in the released NL-ADA8 as we had observed for NL4.3 in the presence of *Mtb*EspR (**Supplementary Figures S2D, E**). With these experiments, we confirmed that mCherry-tagged *Mtb*EspR protein is secreted out from *M.smeg*::*Mtb*EspR and that *Mtb*EspR increases both mycobacterial survival and HIV propagation in the host cells.

### The secreted *Mtb*EspR localizes to the host nucleus during infection

**Figure 3:**
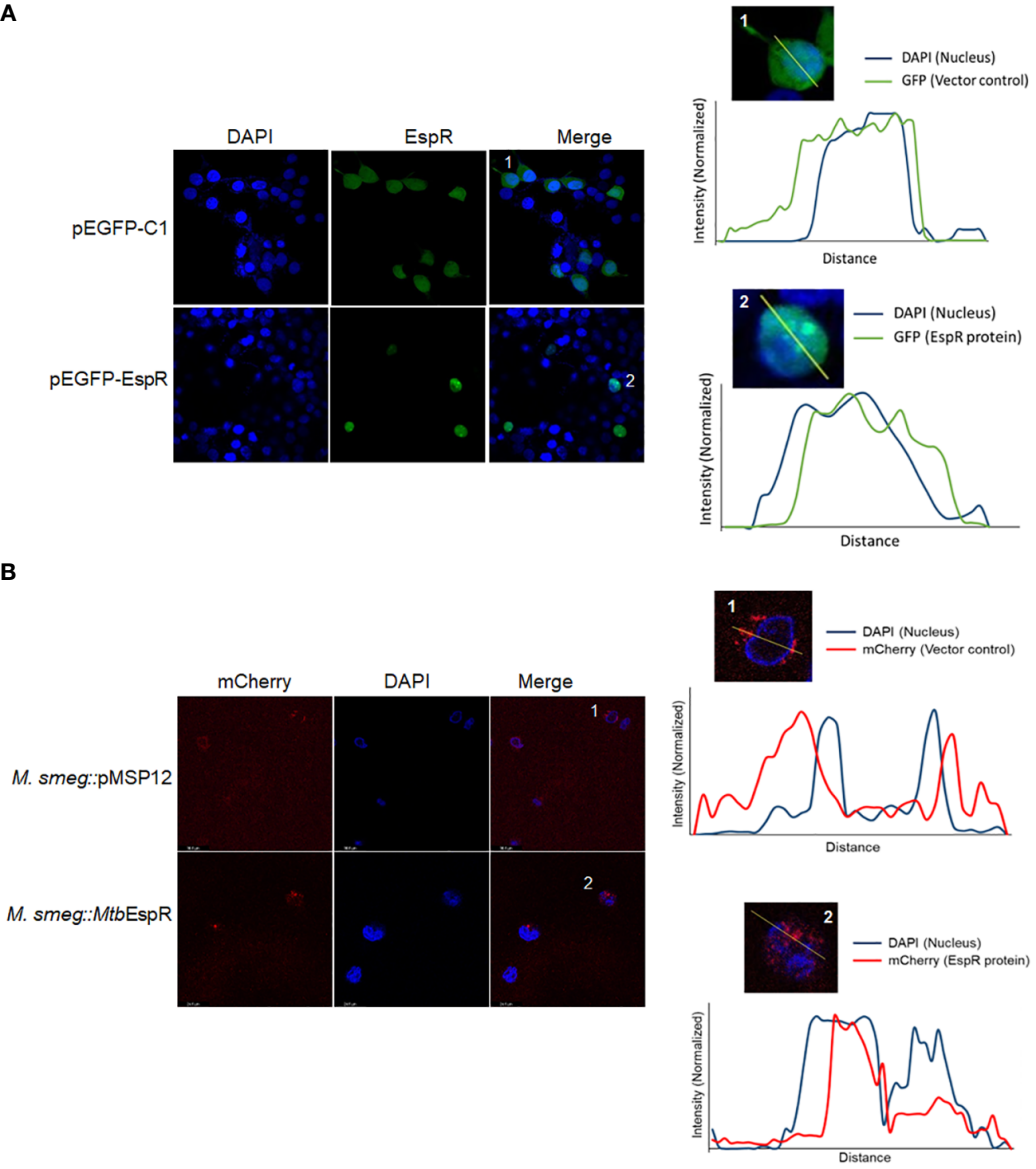
*Mtb*EspR localizes to the host nucleus **(A)** Confocal microscopy of *Mtb*EspR subcellular localization in HEK293T cells upon transfection with pEGFPC1 or pEGFP-EspR, along with plot profiles of representative single-cell images of the insets 1 and 2 were generated using ImageJ software **(B)** Confocal microscopy of *Mtb*EspR localization in THP-1 cells upon infection with *M.smeg*::*pMSP12*, *M.smeg*::*Mtb*EspR. The plot profiles of representative single-cell images of insets 1 and 2 were generated using ImageJ software. All experiments were done at least 3 times.

To comprehend the mechanism of action of *Mtb*EspR and considering that it is a secreted transcription factor, we traced the localization of *Mtb*EspR inside mammalian cells by transient expression of *Mtb*EspR with a GFP tag (pEGFP-EspR) (**Supplementary Figure S3A**) in HEK293T cells and *M.smeg::Mtb*EspR infected THP-1 cells using confocal microscopy by both fixed and live cell imaging. Towards this, we first transfected HEK293T cells with pEGFP-EspR and pC-EspR to identify its subcellular localization by confocal microscopy. We found that *Mtb*EspR localized to the host nucleus as reflected by the cyan color in the merged confocal image (**Figure 3A**; **Supplementary Figure S3B**). The mCherry tagged *Mtb*EspR in *M.smeg::Mtb*EspR infected THP-1 also localized to the nuclei as observed in the merged confocal image and the line plots of representative single cells (**Figure 3B**; **Supplementary Video S3C**). The infection experiments confirmed that *Mtb*EspR is released by *M.smeg::Mtb*EspR and enters the host nucleus. With this, we concluded that *Mtb*EspR enters the host nucleus both upon transient expression and during infection of THP-1 cells.

### *Mtb*EspR binds to the DNA elements in the upstream promoter region of the human IL-4 gene and positively regulates the IL-4 promoter

**Figure 4:**
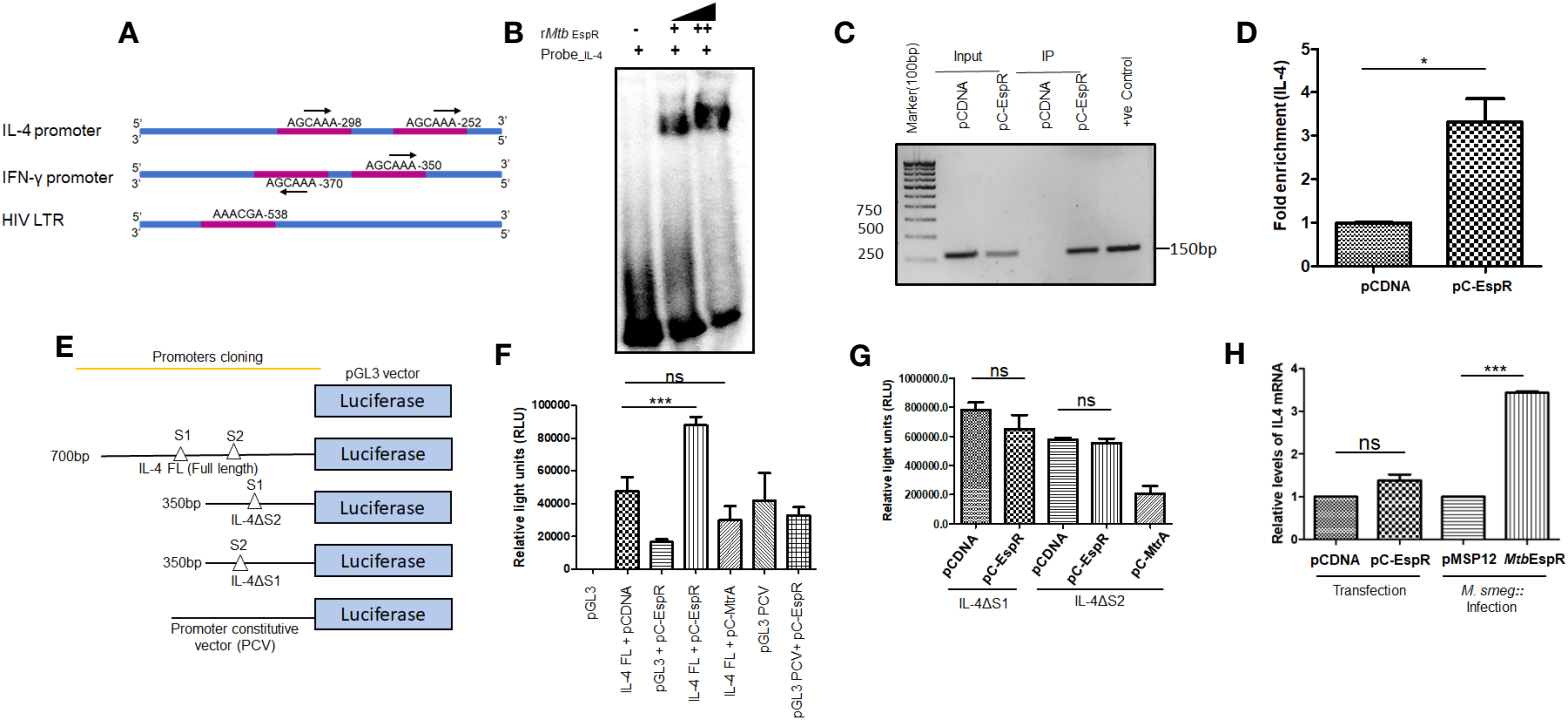
*Mtb*EspR binds and positively regulates the IL-4 promoter **(A)** Schematic representation of the different EspR binding sites in the promoter regions of IL-4, IFN-γ and HIV LTR **(B)** Electrophoretic mobility shift assay depicting the binding of *Mtb*EspR to the IL-4 promoter with increasing concentration of purified r*Mtb* EspR protein **(C)** ChIP assay was performed using an anti-Myc antibody after transfecting with pCDNA or pC-EspR along with pGL3-IL-4 FL reporter followed by PCR using IL-4 promoter-specific primers **(D)** qRT-PCR was performed using IL-4 promoter-specific primers to quantify the occupancy of EspR on the IL-4 promoter region **(E)** Schematic illustration of the various IL-4 promoter constructs used for the luciferase reporter assay **(F)** HEK293T cells were transfected with full-length IL-4 reporter construct either in the presence or absence of *Mtb*EspR (pC-EspR), followed by measurement of luciferase activity 48 h post-transfection. **(G)** The luciferase assay was performed with deletion constructs of IL-4 binding sites (IL-4ΔS1 and IL-4ΔS2 promoters) upon transient expression of *Mtb*EspR (pC-EspR). *Mtb* MtrA was used as a non-specific *Mtb* protein **(H)** qRT-PCR was performed to quantify IL-4 mRNA in the presence and absence of *Mtb*EspR protein either by transient transfection (pC-EspR) or during infection (*M.smeg*::*Mtb*EspR). All experiments were done at least 3 times. The significance was determined using an unpaired student’s t-test. The p-values are denoted as ***p <=0.001 and *p <=0.05, while non-significant values are denoted by n.s.

As mentioned earlier, *Mtb*EspR is a mycobacterial transcription factor that binds to its cognate DNA element for transcriptional regulation (8). With the evidence that it indeed reaches the nucleus of the infected cell and also enhances mycobacterial survival and HIV propagation during co-infection, we next investigated if *Mtb*EspR regulates any of the host gene expressions or HIV LTR. We manually screened for EspR consensus sequences in the promoters of the host innate immune genes as well as HIV LTR. Among the different innate immune gene regulators, we identified the EspR consensus sequence in IL-4 and IFN-γ promoters. We also found an EspR consensus binding site in HIV LTR (**Figure 4A**). Upon analysis of the EspR binding sites, we noted that human IL-4 and IFN-γ upstream regions have two adjacent EspR binding consensus sequences each, but HIV LTR had a single binding site. However, the orientation of the two binding sites is different for IL-4 and IFN-γ, with tandem orientation in the IL-4 promoter region and reverse in the IFN-γ promoter (**Figure 4A**). To confirm the occupancy of these sites by EspR, we performed EMSA using r*Mtb*EspR with the probes of IL-4, IFN-γ promoters, and HIV LTR, wherein we observed that purified recombinant *Mtb*EspR binds to the IL-4 promoter probe (**Figure 4B**), but not to that of IFN-γ or HIV LTR (**Supplementary Figure S4**).

To confirm the protein-DNA interaction of *Mtb*EspR and IL-4 promoter *in vivo*, we performed chromatin immunoprecipitation assay (ChIP)-PCR in HEK293T cells transiently transfected with pC-EspR using anti-Myc antibody and IL-4 specific upstream primers. The ChIP analysis showed an enrichment of the IL-4 promoter in pC-EspR transfected HEK293T cells but not in its vector control cells as visualized by semi-quantitative (**Figure 4C**) and quantitative PCR (**Figure 4D**). Overall, these experiments suggested that *Mtb*EspR binds to the IL-4 promoter both *in vitro* and *in vivo*.

After establishing that *Mtb*EspR binds to the IL-4 promoter, we further investigated if *Mtb*EspR indeed transcriptionally regulates the host IL-4 gene. Towards this, we cloned IL-4 full-length (FL, 700 bp upstream to IL-4 gene TSS), IL-4 ΔS1 (~350 bp upstream to IL-4 gene TSS), and IL-4 ΔS2 promoters (~350 bp upstream to IL-4 gene TSS) in the MCS of pGL3 basic vector (**Figure 4E**). These reporter constructs were transfected in HEK293T cells followed by luciferase assays. To evaluate the effect of *Mtb*EspR, the reporter constructs, pGL3-IL-4 FL or pGL3 PCV (having a constitutive SV40 promoter as non-specific promoter control) were co-transfected with either pC-EspR or its vector control (pCDNA). An increase in luciferase activity (RLU) was observed upon co-transfection of pGL3-IL-4 FL and pC-EspR but not with the control pGL3 PCV signifying a positive regulation of IL-4 in the presence of *Mtb*EspR (**Figure 4F**). The mutant reporter constructs with either S1 or S2 deletions did not influence luciferase activity as compared to respective vector controls, suggesting that both EspR consensus binding sites are required for EspR binding and regulation of the target gene expression (**Figure 4G**). With this, we concluded that *Mtb*EspR is an activator of IL-4 gene expression.

To validate these observations *in vivo*, we monitored IL-4 expression at mRNA level in THP-1 macrophages both upon infection with *M.smeg::Mtb*EspR, and transfection with pC-EspR by qRT-PCR. The results showed an increase in IL-4 mRNA levels both upon infection and transfection as compared to their respective vector controls (**Figure 4H**). Transfection of THP-1 cells with the pC-EspR could only marginally increase IL-4 transcript levels, but a 3-fold increase was observed during infection. These experiments suggested that *Mtb*EspR increased IL-4 gene expression by binding to the upstream of the IL-4 promoter.

### *Mtb*EspR alters the host cytokine environment and macrophage polarization supporting mycobacterial persistence

**Figure 5:**
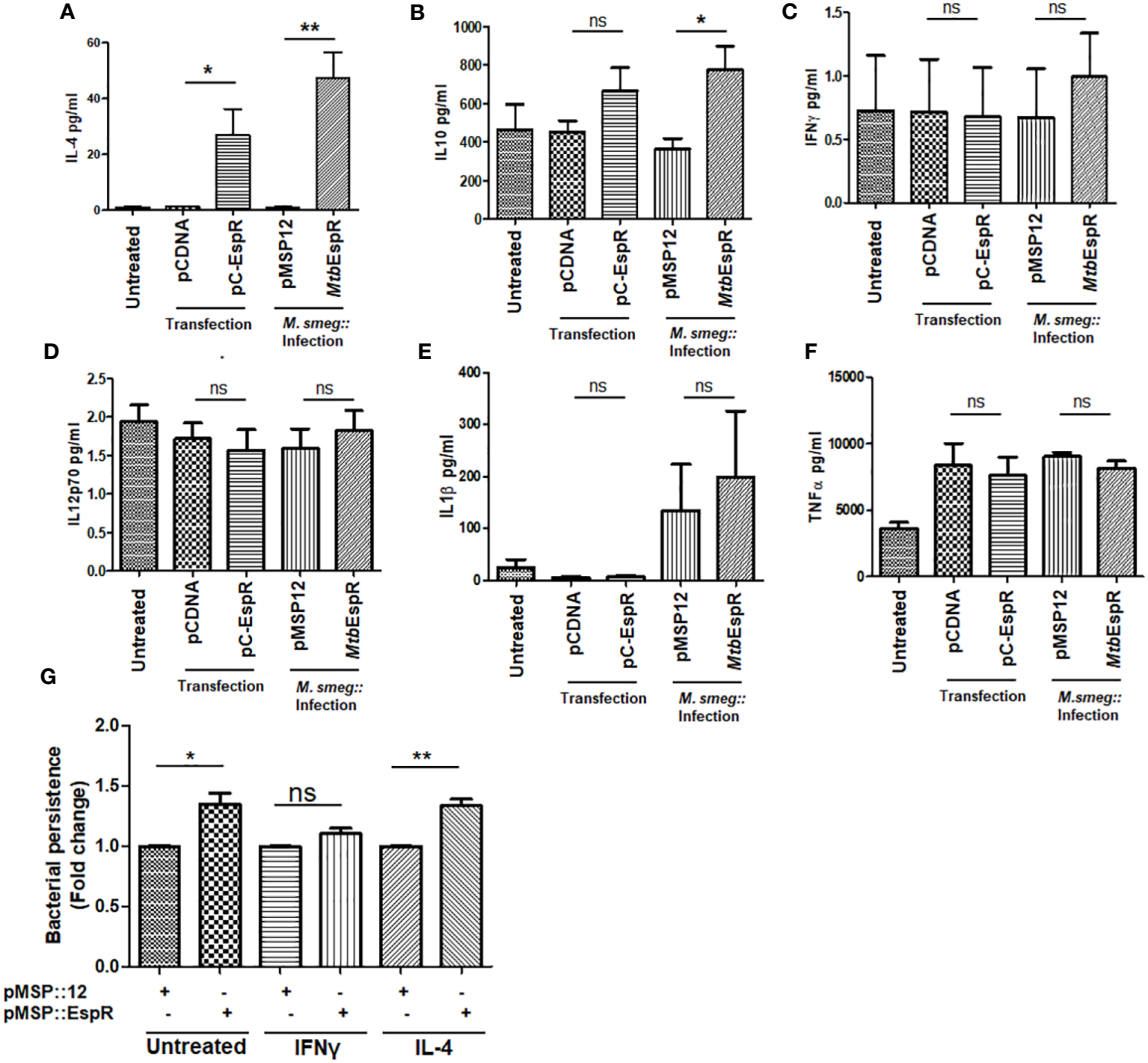
*Mtb*EspR orients the macrophage immune response to Th2-type PMA-treated THP-1 macrophages either transiently expressing *Mtb*EspR (pC-EspR) or infected with *M.smeg*::*Mtb*EspR were evaluated for pro-inflammatory or anti-inflammatory cytokines by ELISA: **(A)** IL-4, **(B)** IL-10, **(C)** IFN-γ, **(D)** IL-12 p70, **(E)** IL-1β, **(F)** TNF-α. **(G)** Bacterial persistence using Alamar Blue assay was analyzed in IFN-γ, and IL-4 treated THP-1 macrophages, after infecting with *M.smeg*::*pMSP12*, *M.smeg*::*Mtb*EspR. All experiments were done at least 3 times. The significance was determined using an unpaired student’s t-test. The p-values are denoted as **p <=0.01; and *p <=0.05, while non-significant values are denoted by n.s.

Having observed that *Mtb*EspR positively regulates the IL-4 promoter and knowing that IL-4 is critical for the shift towards Th2-type immune response, we next investigated the cytokines that are released from THP-1 upon infection with *M.smeg*::*Mtb*EspR and compared that with *M.smeg::pMSP12* infection. We scored for IFN-γ, IL-12 p70, IL-1 β, TNF-α, IL-10, and IL-4 in their culture supernatants upon infection. The analysis clearly showed that there were no differences in levels of the proinflammatory cytokines, TNF-α, IFN-γ, IL-12 p70, and IL-1 β upon either *M.smeg::pMSP12* or *M.smeg*::*Mtb*EspR infections. However, significant differences were noticed in the levels of regulatory cytokines IL-10 and IL-4 (**Figures 5A–F**), wherein *M.smeg::MtbEspR* infection showed higher cytokine titers, clearly pointing to an inclination towards Th2 immune response. We next checked the polarization of THP-1 macrophages upon infection with either *M.smeg::pMSP12* or *M.smeg*::*Mtb*EspR. The polarization of THP-1 macrophages was evaluated by FACS using CD64 and CD206 as M1 and M2 polarization surface markers respectively. THP-1 macrophages stimulated with either IFN-γ or IL-4 were used as positive controls for M1 and M2 polarization (**Supplementary Figures S5A–C**). We observed that transfection of pC-EspR or infection with *M.smeg*::*Mtb*EspR shifted the macrophage population to the M2 state (**Supplementary Figures S5A–C**). To complement these experiments and delineate that the M2 polarization is indeed due to *Mtb*EspR, we repeated the above experiment by transient transfection with pC-EspR. As observed in infection, we saw a similar trend in the levels of secreted cytokines upon transfection (**Figures 5A–F**) as in THP-1 polarization (**Supplementary Figures S5A–D**). To reconfirm that divergent polarized macrophage populations showed differential clearance of *M.smeg*::*Mtb*EspR, we treated THP-1 cells with either IFN-γ or IL-4 before infecting them with *M.smeg::pMSP12* and *M.smeg*::*Mtb*EspR (**Figure 5G**). As evident from the plots in **Figure 5G**, we observed an increased load of *M.smeg*::*Mtb*EspR as compared to *M.smeg::pMSP12* upon IL-4 treatment. We also observed that *M.smeg*::*Mtb*EspR marginally survived better even in IFN-γ treated THP-1, but the experiments were not statistically significant. The control experiments with *M.smeg::pMSP12* infection alone are shown in **Supplementary Figure S5E**. With these experiments, we concluded that *Mtb*EspR-mediated alterations in cytokine microenvironment to Th2-type promoted the increased survival of *M.smeg::MtbEspR*.

**Figure 6:**
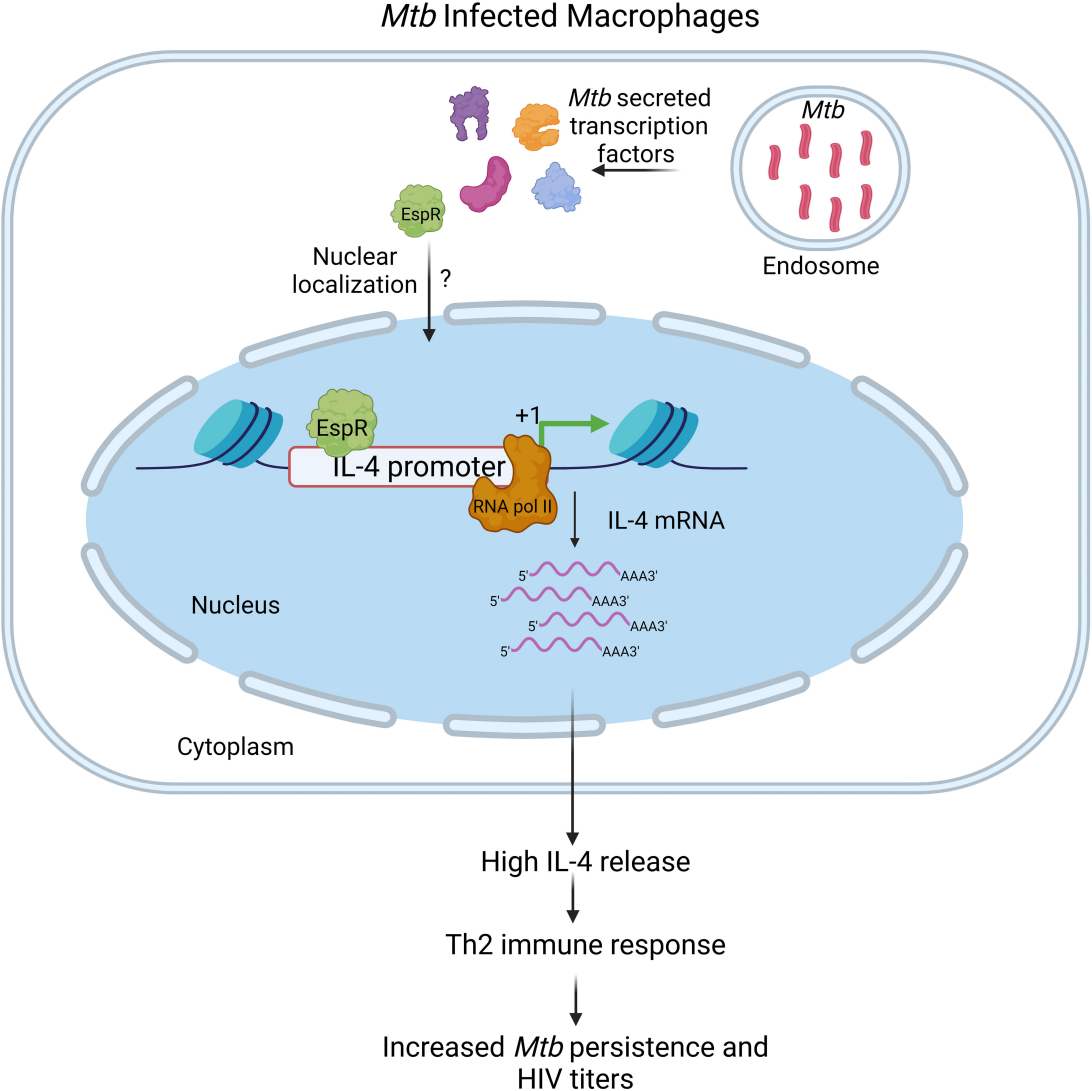
Schematic representation of the role of *Mtb*EspR in *Mycobacterium tuberculosis* persistence *Mycobacterium tuberculosis* infects macrophages, where they primarily multiply and persist. Although the host exerts several innate defense mechanisms, they are thwarted by various mycobacterial proteins, either surface localized or secreted that have a major role in host-pathogen interaction. *Mtb*EspR is one of the secreted proteins in response to microbicidal stress conditions, that enters the host nucleus, wherein it interacts with host DNA elements such as IL-4 and activates the host IL-4 gene expression and ultimately increasing IL-4 cytokine levels, shifting the immune response of the host from Th1- to Th2-type that is conducive for mycobacterial as well as viral multiplication. Created with Biorender.com.

Conclusively, this study shows that the *Mtb* factor EspR is secreted during both standard growth conditions as well as microbicidal stresses and infection, localizes to the nucleus of the infected host cell, and binds to the IL-4 promoter, augmenting its expression, and thereby enhancing secreted IL-4 levels (**Figure 6**). In summary, this shifts the cytokine microenvironment to a Th2-type immune response enhancing mycobacterial persistence and HIV propagation both during single infection and during HIV co-infection.

## Discussion

*Mtb* is a remarkably successful pathogen as it effectively thwarts host defenses through a multitude of mechanisms owing to its secreted proteins and metabolites. In the present study, we have revealed three new facets of *Mtb*EspR in host-pathogen interaction (1). *Mtb*EspR gains access to the host nucleus and directly regulates the host innate immune genes. (2) Mechanistic details of *Mtb*EspR mediated shift in the Th1 and Th2 microenvironment. (3) *Mtb*EspR positively regulates HIV propagation during co-infection.

Secretion of transcriptional regulator proteins from pathogens and their binding to host DNA elements is extremely rare in biology, with a few examples from viruses. For example, HIV Tat, a transcription factor, released from an infected cell can reach the nucleus of a bystander host cell and interact with DNA (37, 38). *Mtb*EspR from this study represents a transcription factor from pathogenic mycobacteria that showed the capacity to alter the expression of a host gene by directly binding to the host DNA elements. While there is a study that reported that EspR is not secreted by *Mtb* (9), this study and others have shown that EspR is secreted (10, 39). It was also intriguing to note that as expected out of a transcription factor, *Mtb*EspR indeed entered the host nucleus. Most proteins that are targeted to the nucleus possess a consensus nuclear localization signal (NLS) for nuclear entry. *Mtb*EspR, however, lacks a conventional NLS and hence can be speculated to be using specific host nuclear import machinery to gain entry into the nucleus during infection. *Mtb*EspR is a small protein and hence it may also gain access to the nucleus through nuclear pore complexes by simple diffusion. This may be addressed by exploring the host protein interaction network of *Mtb*EspR during infection.

With the availability of the EspR binding sequence (8), we scanned the promoter regions of many human cytokine genes. Among them, IL-4 and IFN-γ gene upstream sequences showed two consensus EspR binding sites. Our earlier reports have shown that these two cytokines are concurrently upregulated in HIV-TB co-infected patients (18). Hence, we also scanned the HIV LTR sequence and observed the presence of a single EspR binding site. The orientation of EspR binding sites in the promoters of IL-4 and IFN-γ are different. While the repeats are tandem in IL-4, the orientation of the two EspR binding sequences are in reverse orientation in IFN-γ. With EMSA and ChIP data clearly showing that EspR binds to IL-4, but not to IFN-γ or HIV LTR sites, one can speculate that two tandem binding sequences are important for EspR-DNA interactions, although in-depth biochemistry and characterization of the DNA binding property of EspR is required. The reporter assays showed that EspR is an activator of the IL-4 promoter. We also noted that EspR needs two consensus-binding motifs for the regulation of transcription. When we deleted either of the consensus sequence from the IL-4 promoter and performed the reporter assays, we could not observe any significant enhancement in luciferase activity upon EspR expression, suggesting that EspR needs both the consensus sequences for the transcriptional regulation of the IL-4 promoter.

IL-4 is an anti-inflammatory cytokine and activates the Th2-type immune response and it also reciprocally regulates the Th1-type immune response. However, *Mtb* infection in macrophages causes a biphasic polarization with an early activation to the M1 polarization state (Th1-type immune response) and is soon shifted to an alternately polarized and compromised repair state M2 (Th2-type immune response) (40). With *Mtb*EspR upregulating the expression of IL-4, one would expect that it will orient the response to the Th2-type. In our analysis of cytokine profile in the presence of *Mtb*EspR, Th2 cytokines were dominant. Such observations have been made for many *Mtb* proteins, such as ZMP-1 (22), HupB (41), and PPE34 (42). These studies, although have indicated that many *Mtb* proteins skew the host responses to Th2-type, no mycobacterial factor has been shown to directly regulate the expression of Th2 cytokines by binding to host DNA elements.

Most intracellular infections require dominant Th1-type cytokine-induced cell-mediated responses to clear the pathogen. When we observed that *Mtb*EspR is orienting the immune environment to the Th2-type, we speculated that it will also compromise the host cell to clear other intracellular pathogens associated with *Mtb* co-infections. As *Mtb*-HIV is one of the most common co-infections, we studied the impact of *Mtb*EspR on the survival of both mycobacteria and HIV during co-infection. HIV is a known risk factor for developing into active TB, which compromises the immune system by depleting the CD4^+^T cells and accelerating the progression of TB (43). Simultaneously, HIV titers also increase during co-infection with TB (44, 45). *Mtb* can also make the cellular environment more conducive to HIV propagation by increasing the expression of host factors that are positive regulators of HIV LTR (7, 46). However, *Mtb*EspR could not directly interact with HIV LTR, but the viral propagation increased in the presence of EspR over-expressing mycobacterial strain. With our study clearly showing that EspR does not directly bind to HIV LTR to influence HIV replication, we speculate that the bystander effect of EspR is through its impact on increased secretion of IL-4 by infected macrophages, which in turn generates a conducive environment for HIV replication. IL-4-mediated effect on HIV can be either stimulatory or inhibitory and is context-dependent. Such as treatment with IL-4 has been experimentally shown to stimulate HIV production by infected monocytes and macrophages (47). Further, Mikovits et al, reported that pre-treatment of peripheral monocytes for 48-72 h with IL-4 increased acute HIV infection, and similar effects were seen when the U1 and R-THP-1 monocytoid cell lines (the cell lines with restricted HIV expression) were treated with IL-4 (48). Another study showed that IL-4 stimulation enhanced transcription that is mediated by IL-4 stimulated NF-κB nuclear translocation. Further, they also state that IL-4 and IL-13 inhibited HIV replication at the transcriptional level in monocyte-derived macrophages after 3 to 5 days of adherence to the surface of plastic culture plates (49). Interestingly, it has been observed that IL-4 favored the replication of T-tropic (X4) virus as compared to M-tropic (R5) replication (50), favoring X4 virus generation. One may note that secondary infections like *Mtb* or other infections, are more frequent at chronic stages. It is known that during chronic stages of HIV infection, the M-tropic viruses (R5) are low and T-tropic viruses (X4), and dual tropic viruses (R5/X4) are predominant (51). Further, it is also indicated that *Mtb* induces the shift of the R5 virus to the X4 virus by increasing the expression of CXCR4 receptor (52). This suggests that mycobacteria-induced selection of X4 virus may also be mediated via altering IL-4 levels. In addition, that *Mtb*EspR over-expression in HEK293T cells augmented propagation of both NL4.3 and NL-ADA8, supported the notion that increase in viral propagation by *Mtb*EspR does not solely depend upon IL-4 secretion. It is reported that *Mtb*EspR inhibits apoptosis in the macrophage cells (11). Such phenomena (inhibition of apoptosis) are supportive for active HIV replication (53). Therefore, we speculate that EspR enhances the HIV replication by several direct or indirect mechanisms, such as inhibiting apoptosis and promoting Th2-type microenvironment. *Mtb*EspR interactome in host macrophages should be investigated to discover additional mechanisms.

In summary, in this study, we found that secreted mycobacterial protein EspR regulates host IL-4 cytokine levels, which skews the immune response to Th2-type leading to immune evasion. Our study shows for the first time that a bacterial transcription factor directly regulates the host gene transcription. This data provides new molecular insights into the mycobacterial protein-mediated regulation of the host genome and HIV propagation, adding to our understanding of mycobacterial infection and co-infection biology that can be used for elucidating new targets for therapeutic interventions aimed at controlling TB or HIV-TB co-infection.

## Data availability statement

The original contributions presented in the study are included in the article/**Supplementary Material**. Further inquiries can be directed to the corresponding author.

## Author contributions

SY: Formal Analysis, Writing – original draft, Writing – review & editing, Data curation, Investigation, Methodology, Validation, Visualization. AA: Formal Analysis, Investigation, Methodology, Writing – review & editing. AC: Formal Analysis, Investigation, Methodology, Writing – review & editing. SS: Formal Analysis, Investigation, Methodology, Writing – review & editing. KM: Formal Analysis, Investigation, Methodology, Writing – review & editing. SB: Formal Analysis, Writing – review & editing, Conceptualization, Funding acquisition, Project administration, Resources, Supervision, Writing – original draft.
