# Supplementary material for: Mycobacterium tuberculosis EspR modulates Th1-Th2 shift by transcriptionally regulating IL-4, steering increased mycobacterial persistence and HIV propagation during co-infection
Source: Front Immunol. 2023 Oct 19;14:1276817. doi: 10.3389/fimmu.2023.1276817 (PMC10621737; doi:10.3389/fimmu.2023.1276817)
Supplement: Supplementary file 1 [file DataSheet_1.docx]

***Mycobacterium tuberculosis* EspR modulates Th1-Th2 shift by transcriptionally regulating IL-4, steering increased mycobacterial persistence and HIV propagation during co-infection**

Sriram Yandrapally, Anushka Agarwal^#^, Archismita Chatterjee^#$^, Satarupa Sarkar^†,^ Krishnaveni Mohareer^†^, and Sharmistha Banerjee*

Department of Biochemistry, University of Hyderabad, Hyderabad, India.

^#^ these authors contributed equally to this work

^†^ these authors contributed equally to this work

^$^Department of Cellular and Molecular Neuroscience, National Brain Research Centre (NBRC) Manesar, Gurgaon, India.

*Address for correspondence: [sbsl@uohyd.ac.in](mailto:sbsl@uohyd.ac.in)

Table S1: Primers used in this study

| pET28a/pCDNA3.1-EspR | FP | 5’ ACGGATCCATGAGCACGACGTTCGCTG 3’ |
| --- | --- | --- |
|  | RP | 5’CCCTCGAGAGCGTCGATCCCTTCGGC 3’ |
| pEGFPc1 EspR | FP | 5’CCCAAGCTTCGATGAGCACGACGTTCGCTGCC3’ |
|  | RP | 5’CGGGATCCAGCGTCGATCCCTTCGGCACG3’ |
| pMSP12 EspR | FP | 5’GTAAGCTTATGAGCACGACGTTCGCTG 3’ |
|  | RP | 5’GAGGTACCAGCGTCGATCCCTTCGGCAC 3’ |
| pGL-3 IL4 Full | FP | 5’ AACTCGAGGGGACCCAAACTAGGCCTCAC3’ |
|  | RP | 5’ GGAAGCTTAGAACAGAGGGGGAAGCAGTTGG 3’ |
| pGL-3 DS2 IL4 | FP | 5’ AACTCGAGGGGACCCAAACTAGGCCTCAC3’ |
|  | RP | 5’ GGAAGCTTATTAATCTTATCAGCTTGGGGCTGC 3’ |
| pGL-3 DS1 IL4 | FP | 5’ AACTCGAGCAGCAGCAGCCCCAAGCTGATA 3’ |
|  | RP | 5’ GGAAGCTTAGAACAGAGGGGGAAGCAGTTGG 3’ |
| qPCR IL4 | FP | 5’ CTGTGCACCGAGTTGACCGTAACAG 3’ |
|  | RP | 5, AGAGGTTCCTGTCGAGCCGTTTCA3’ |
| qPCR EspR | FP | 5’CGCTCAGGAAACCGTACGAACCC3’ |
|  | RP | 5’AACACCTTCTGCTGCGCCGCG 3’ |
| IL4 ChiP | FP | 5’GACACCTGTGGCCTCTCCCTTC3’ |
|  | RP | 5’AAAGTTTCAGCATAGGAAATTACACCATA3’ |
| IL4 full EMSA | TS | 5’CTATGCAAAGCAAAAAGCCAGCAGCAGCCCCAAGCTG  ATAAGATTAATCTAAAGAGCAAATTATGGTG3’ |
|  | BS | 5’CACCATAATTTGCTCTTTAGATTAATCTTATCAGCTTGG  GGCTGCTGCTGGCTTTTTGCTTTGCATAG3’ |
| IFNγ EMSA | TS | 5’GAATTTCGTTTTTCACTTGTTCCCAACCACAAGCAAATGATCAATGT3’ |
|  | BS | 5’ACATTGATCATTTGCTTGTGGTTGGGAACAAGTGAAAAACGAAATTC3’ |
| HIV LTR EMSA | TS | 5’CAGTTGAACCAGAGCAAGTAGAAGAGGCC3’ |
|  | BS | 5’GGCCTCTTCTACTTGCTCTGGTTCAACTG3’ |
| DS2 IL4 | TS | 5’TAATCTAAAGAGCAAATTATGGTGTA3’ |
|  | BS | 5’TACACCATAATTTGCTCTTTAGATTA3’ |
| DS1 IL4 | TS | 5’TTCTATGCAAAGCAAAAAGCCAGCAG3’ |
|  | BS | 5’CTGCTGGCTTTTTGCTTTGCATAGAA3’ |

TS- Top strand, BS- Bottom strand

**Supplementary Figure S1**


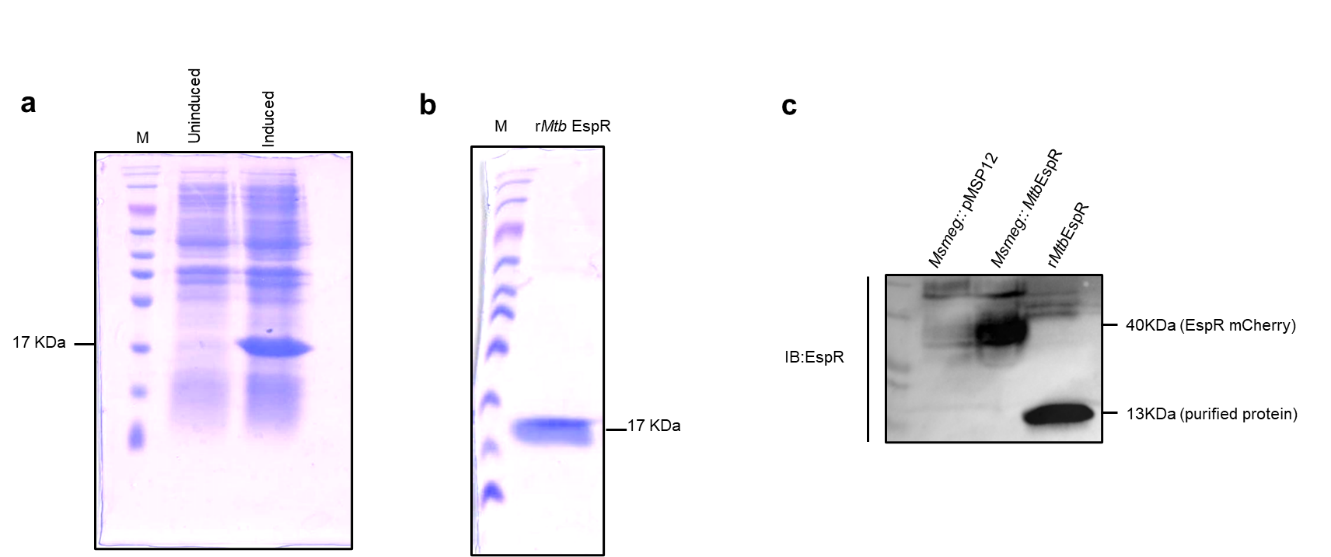


**Supplementary Figure S1**: (a) SDS PAGE showing the expression of r*Mtb*EspR protein in the BL21 DE3 cells transformed with pET-*Mtb*EspR after induction with 1mM IPTG for 4 h. (b) r*Mtb*EspR protein was purified from native condition from pET28-*Mtb*EspR transformed BL21 DE3 cells by affinity chromatography. (c) EspR antibody containing serum was used to probe *Mtb*EspR from *M.smeg*::*pMSP12* and *M.smeg::Mtb*EspR cell lysates by immunoblotting.

**Supplementary Figure S2**

**
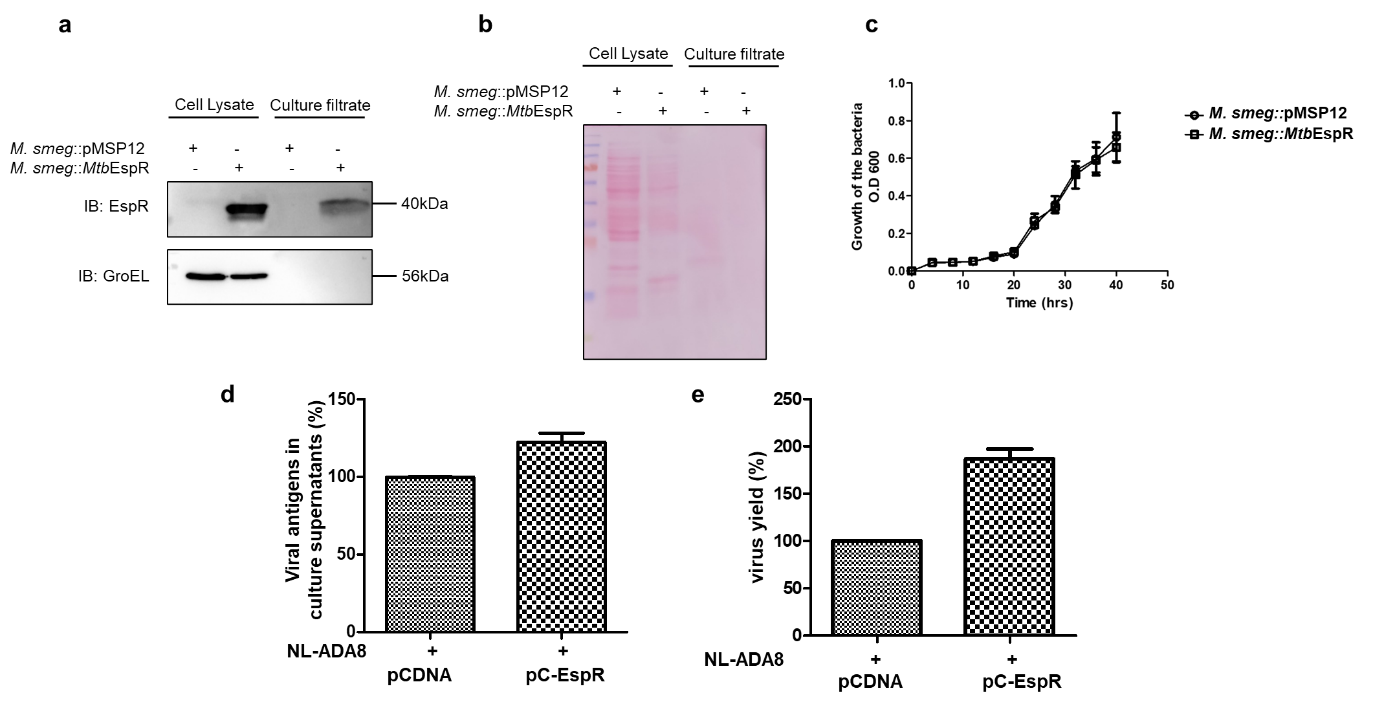
**

**Supplementary Figure S2**: (a) Immunoblot showing mCherry tagged *Mtb*EspR secretion from *M.smeg*::*MtbEspR* in *in vitro* growth culture. GroEL was used as a cell lysis control. (b) Ponceau staining of the blot as loading control for the experiment shown in (a). (c) Growth kinetics of *M.smeg*::pMSP12 and *M.smeg::MtbEspR* in *in vitro* standard growth media. The O.D was measured at 600nm. (d) Relative percentage of the released viral antigens (NL-ADA8) in the culture supernatant as determined by ELISA in pCDNA, pC-EspR transfected HEK293T cells. (e) Infectious virus yield by TZM-bl assay. Briefly, equal volumes of the culture supernatants were used for infection and the luciferase assay was performed 24 h post infection. The NL-ADA8 present in the culture supernatant of pC-EspR transfected cells resulted in higher luciferase values than those collected from the culture supernatants of pCDNA, suggesting an overall increase in the virus yield in the presence of *Mtb*EspR.

**Supplementary Figure S3**


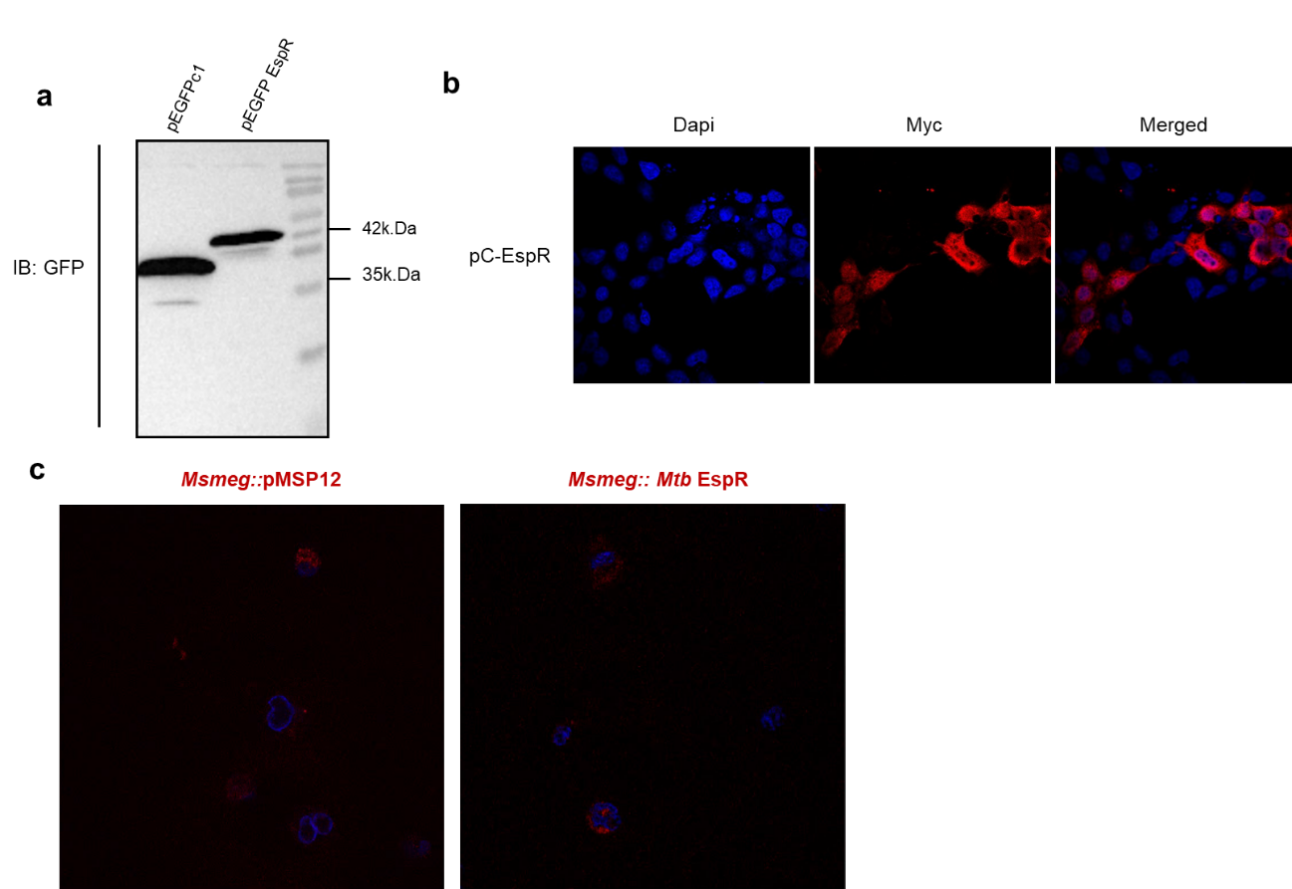


**Supplementary Figure S3:** (a) Immunoblot showing the expression of *Mtb*EspR protein tagged with GFP using anti-GFP antibody. (b) Confocal microscopy of *Mtb*EspR subcellular localization in HEK293T cells upon transfection with pC-EspR. (c) Live cell imaging showing the subcellular localization of *Mtb*EspR post infection of THP-1 macrophages with *M.smeg*::*pMSP12* or *M.smeg::Mtb*EspR. All experiments were done at least 3 times.

**Supplementary Figure S4**


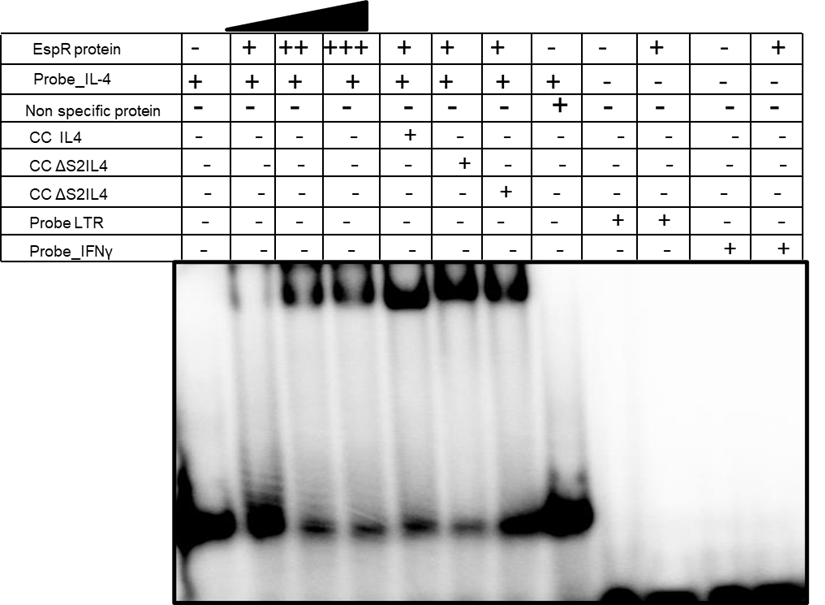


**Supplementary Figure S4**: Electrophoretic mobility shift assays were performed with the purified r*Mtb*EspR using either the human IL-4 promoter, or IFN-γ promoter or HIV LTR probes. IL-4 promoter binding was competed with 100X of self or ΔS1 or ΔS2 motifs. All experiments were done at least 3 times.

**Supplementary Figure S5**


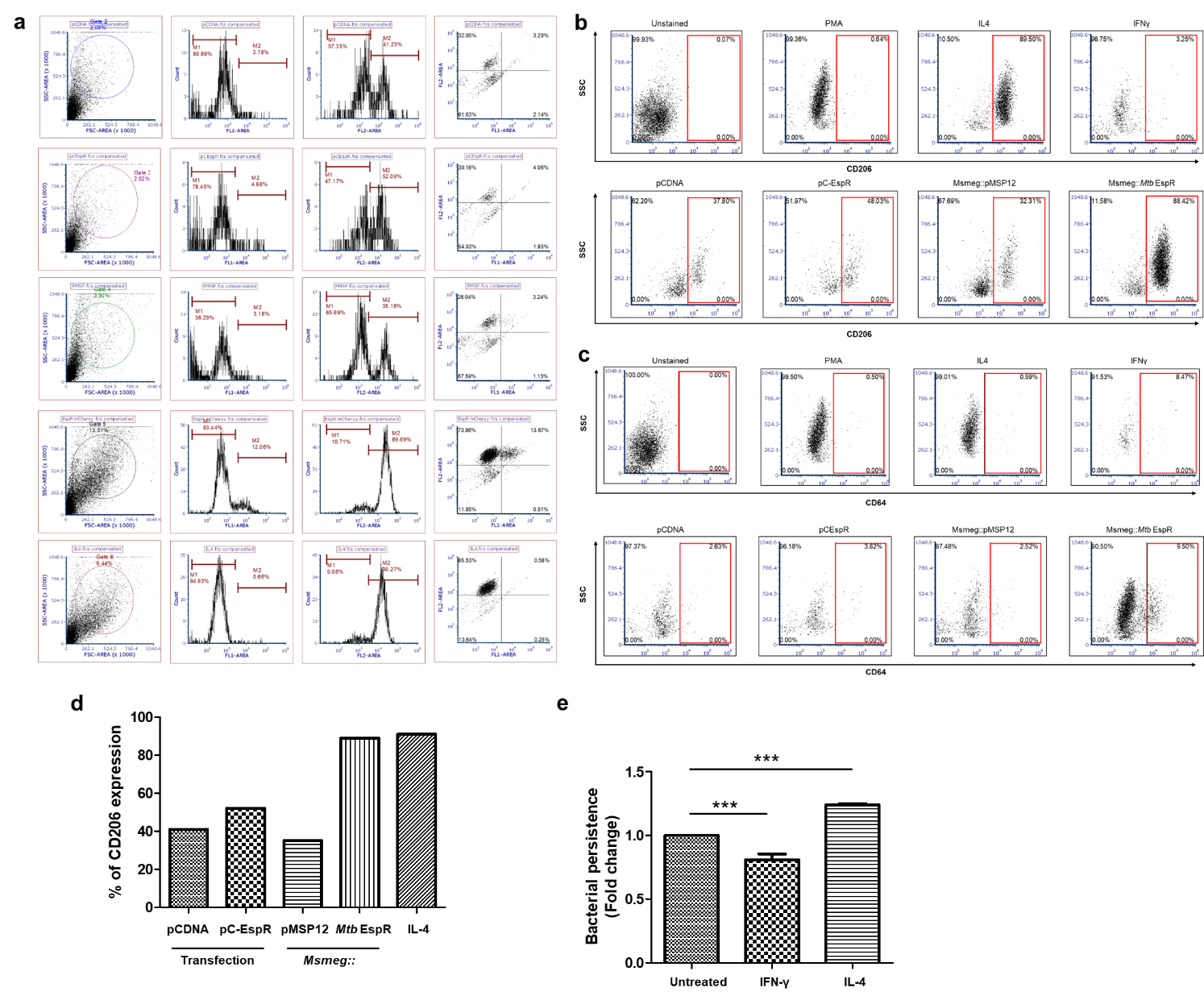


**Supplementary Figure S5**: (a-c) Flow cytometric analysis was performed upon transient expression of *Mtb*EspR (pC-EspR) or infection with *M.smeg::Mtb*EspR by evaluating CD64 (M1 or classically activated macrophage marker) and CD-206 (M2 or alternatively activated macrophage marker) markers on the cell surface of macrophages. IFN-γ treatment served as a positive control for M1 polarization and IL-4 treatment for M2 polarization. (d) Quantification of the CD206 protein expression on cell surface for experiment represented in a. (e) Fold change in bacterial persistence using Alamar blue assay in IFN-γ and IL-4 treated THP-1 macrophages post infection with *M.smeg*::*pMSP12*. These experiments were done once with technical replicates. The significance is determined using an unpaired student’s t-test. The p-value denotes *** p <=0.001.
